# Rhegmatogenous retinal detachment in Scotland: research design and methodology

**DOI:** 10.1186/1471-2415-9-2

**Published:** 2009-03-24

**Authors:** Danny Mitry, David G Charteris, David Yorston, Brian W Fleck, Alan Wright, Harry Campbell, Jaswinder Singh

**Affiliations:** 1 Princess Alexandra Eye Pavilion, Chalmers Street, Edinburgh, UK; 2 Department of Public Health Sciences, University of Edinburgh, Edinburgh, UK; 3 Moorfields Eye Hospital, City Road, London, UK; 4 Gartnavel General Hospital, Great Western Road, Glasgow, UK; 5 Medical Research Council, Human Genetics Unit, Edinburgh, UK

## Abstract

**Background:**

Rhegmatogenous retinal detachment (RRD) is a potentially blinding condition and a common cause of ocular morbidity. Establishing an accurate estimate of disease incidence and distribution is an important first step in assessing the healthcare burden related to this condition and in subsequent planning and provision of treatment strategies. The aim of this study is to obtain a first estimate incidence of RRD in Scotland, to estimate the incidence of familial RRD and to describe the known associations of RRD within the study population.

**Methods/Design:**

We have established a national prospective observational study seeking to identify and recruit all incident cases of RRD in the Scottish population over a 2 year period. After fully informed consent, all participants will have a blood sample taken and a full medical history and clinical examination performed including visual acuity, refraction, slit-lamp examination, intra-ocular pressure measurement and detailed fundal examination. We describe the study design and protocol.

**Conclusion:**

This study will provide the first estimate of the annual incidence of RRD in Scotland. The findings of this study will be important in estimating the burden of disease and in the planning of future health care policy related to this condition. This study will also establish a genetic resource for a genome wide association study to investigate if certain genetic variants predispose to RRD.

## Background

Rhegmatogenous retinal detachment (RRD) is a potentially blinding ophthalmic pathology caused by a separation of the neurosensory retina (NSR) from the underlying retinal pigment epithelium and the accumulation of fluid within this potential space. It is responsible for up to 2% of blind and partial sight registrations in Ireland, Scotland and south west England [1-3]. The mainstay of RRD treatment is surgical, accounting for an important proportion of ophthalmic hospital in-patient admissions at an annual cost of over £1.3 million in the U.K [4]. The principles defining an epidemiology study are the careful delineation of the study population from which cases are derived and the effort to ascertain every eligible case from the study population [5]. To date there have been no large-scale systematic epidemiology studies of RRD in Scotland. Previous population based estimates of the incidence of RRD have varied considerably, with overall annual incidence rates reported between 5–17.9 per 100,000 of population [6-16]. Based on these previous reports, we estimate that there are between 500–600 new cases of RRD in Scotland annually.

The genetic risk of RRD has been investigated through family linkage studies[17,18] and twin reports [19-21]. It has also been noted that familial occurrence of RRD is a risk factor for its' development, with a risk ratio of 2.6 for cumulative lifetime risk of RRD in relatives of subjects with RRD compared to relatives of those without RRD[22]. Recently, genome-wide association (GWAS) studies have become established as powerful tools in identifying common genetic variants that predispose to common, complex disease. These case-control studies seek to characterize DNA nucleotide polymorphisms in a large number of cases and disease-free controls to determine if certain polymorphisms are associated with the disease phenotype[23]. To date no GWAS have been conducted on rhegmatogenous retinal detachment. Our study will establish an important resource for further exploration of the genetic aetiology of this condition.

## Aims

### 1 – Epidemiology

#### Primary

To establish a complete case register of new cases of RRD in the Scottish population over a 2 year period

To calculate the incidence of RRD in the Scottish population.

To determine the incidence of familial rhegmatogenous retinal detachment in Scotland.

To examine the prevalence of myopia, trauma, peripheral retinal degeneration and previous intra-ocular surgery within the study sample

#### Secondary

To provide data to inform the planning of vitreoretinal surgical services in Scotland

To define abnormalities present in the second eye of patients presenting with rhegmatogenous retinal detachment.

### 2 – Genome – Wide Association

To utilise the Scottish rhegmatogenous retinal detachment study database in a case-control analysis to identify common genetic variants predisposing to rhegmatogenous retinal detachment.

To conduct a genome-wide association study of RRD to identify novel common genetic variants associated with risk of RRD.

To verify the contribution of these genetic loci in a replication study in collaboration with other well-characterised RRD case-control collections.

## Methods/Design

### Scottish Population

This study is a prospective population-based epidemiology study in Scotland. The study participants are derived from the entire resident population of Scotland. The last formal census was conducted in April 2001 and was administered over one day. Data was gathered on all usual residents (excluding visitors) in Scotland. It was a highly accurate census with an overall response rate of 96% and an estimated coverage of 100%. The usual resident population of Scotland in 2001 was 5.062 million people.

**Figure 1:**
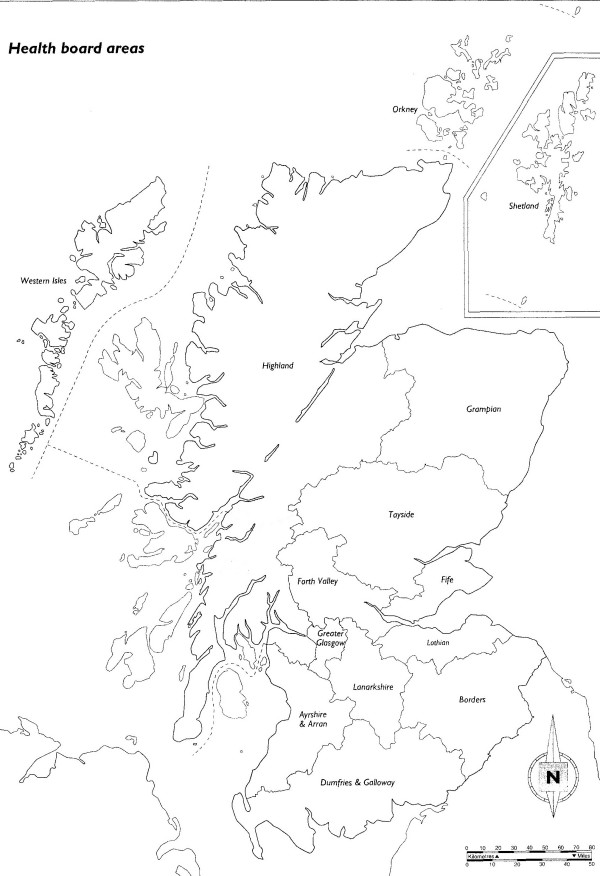
**Health board distribution in Scotland**. (Courtesy of the National Registry Office, Scotland).

**Figure 2:**
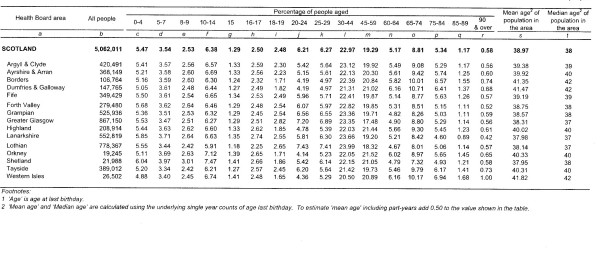
**The age and population structure of all Scottish residents in each health board region in the 2001 population census**. (Courtesy of the National Regsitry Office, Scotland) Note- Argyll and Clyde is now considered part of the Highland Health Board region.

Fourteen health boards are established in Scotland to administer the Scottish health service. These health boards are divided geographically (See Figure 1) and all diagnosed RRD cases in Scotland are referred to one of six vitreo-retinal surgical sites for assessment and surgery. The established structure of the Scottish health service and the organisation of the ophthalmology community in Scotland will permit high levels of co-operation and case ascertainment. Figure 2 highlights the health board distribution and age structure of the Scottish population.

### Participating centres and protocol

The six vireo-retinal surgical sites (Ayr, Glasgow, Edinburgh, Dundee, Aberdeen and Inverness) are geographically distributed across different health boards and they are responsible for and manage the operative workload of cases requiring retinal surgery throughout Scotland. Thus, each identified case will be referred to one of these centres for assessment and further management. There are 16 vitreo-retinal consultant specialists in Scotland who are actively participating in this study.

### Sample Size

**Figure 3:**
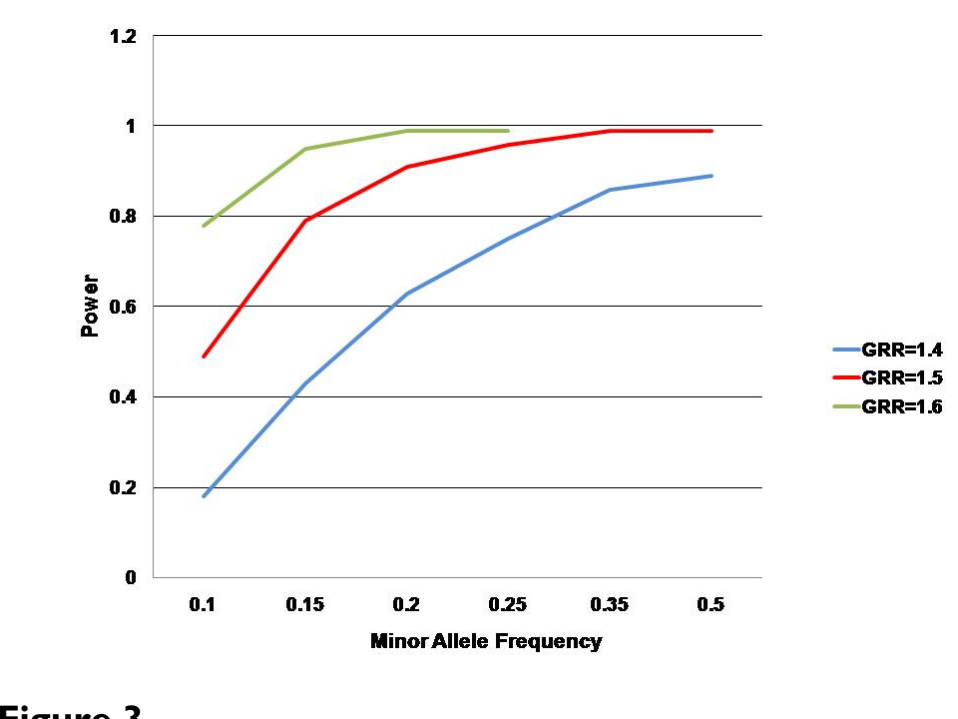
**Power estimates by genotypic relative risk (GRR) and allele frequency for a 2 stage genome wide association study with a total sample size of 2000 cases and controls**.

Based on previous estimates of RRD incidence and current population data, we hypothesise that there are approximately 500–600 new cases of RRD in Scotland annually. A collection period of 2 years with a predicted recruitment rate of 90% will provide a feasible collection period and an appropriate sample size to estimate disease incidence with a 95% confidence interval of less than 3 cases annually per 100,000 of population. (based on Poisson distribution) Moreover, a sample size of 1,000 Scottish cases in conjunction with a further 1,000 cases recruited from elsewhere in the UK and Europe will provide the genetic resource for a 2-stage genome wide association study with acceptable power to detect a genotypic risk ratio of approximately 1.5 at a minor allele frequency of 10–15% or greater. Figure 3 illustrates a number of power calculations for several of different sample sizes with an estimated disease prevalence of 0.003[9] using a case-control power calculator for a two stage association study: 

### Case identification and recruitment

All incident cases of rhegmatogenous retinal detachment presenting to all participating centres are invited for inclusion into the study. The diagnosis of RRD is based on a case definition of "a full thickness break in the neurosensory retina with a surrounding area of sub-retinal fluid extending greater than 2 disc diameters" [7,8,14,24]. No distinction is made in this study between clinical and subclinical retinal detachment, all diagnosed cases are invited[24]. As the original definition shows observer dependant bias, we extended RRD classification in surgical terms as any break in the neurosensory retina with surrounding sub-retinal fluid requiring intervention more than cryotherapy and/or laser treatment to reattach or stabilise the retina. Thus in practice any patient who has a RRD of significant size which is treated with barrier laser alone as well as those who undergo an operative procedure including vitrectomy and internal tamponade, pneumoretinopexy or external buckle are included. Patients are also included if they present with a RRD but for medical, social or occupational reasons the decision to perform surgery was not taken. Blunt traumatic cases of RRD are included, penetrating trauma is excluded. All other types of retinal detachment (exudative, tractional and combined) are excluded. Re-detachments regardless of duration of attachment post-operatively are excluded.

### Data Quality

A number of procedures were established to ensure optimal data quality. Firstly, each centre was visited and didactic tutorials were established with the retinal department staff and nominated local investigators. All nominated investigators were trained by demonstration and explanatory protocols on accurate completion of the questionnaires. Each site is visited regularly to guarantee data completeness. The busier centres are visited between one to three times weekly, and the outlying centres are visited more infrequently, approximately every 2–3 weeks. The principal investigator liaises with all nominated investigators in these outlying sites on a weekly basis. All incident cases are identified and approached for inclusion while in hospital.

### Participant Consent and Data Fields Recorded

Cases eligible for recruitment are confirmed through clinical examination by a retinal consultant. Detailed history and examination is performed on all cases by the principal investigator or by an appointed local investigator. Blood sampling is performed on all consenting participants. Individual informed consent is received on the basis that each participant understands and has time to consider the merits of study participation; participation is voluntary and does not affect subsequent care of the patient; the DNA sample and clinical data about the individual would be stored by the research team at Edinburgh University for use in future research and may be shared with other medical research groups (with appropriate ethical approvals first being obtained where necessary). Data fields gathered comprise:

#### 1. Ocular parameters

History of trauma and type of trauma; refractive status (prior to cataract or retinal surgery); previous ophthalmic history; including date of any previous ophthalmic surgery or laser treatment; nature and duration of symptoms; route of referral; type of retinal detachment at presentation; vitreous – attached/detached, syneresis, features of Sticklers/Wagners syndrome or other hereditary vitreo-retinopathy; peripheral retina – Presence and subtype of any peripheral retinal degeneration; second eye – documentation of any vitreo-retinal pathology.

#### 2. Personal, family history, demographic, treatment

Ethnicity; parental consanguinity; family history of eye disease; family history of retinal detachment. A positive family history is considered to be any first degree relative affected by a retinal detachment which required treatment. (i.e. subclinical cases which did not warrant treatment were not considered) Demographic data is taken direct from patients and NHS clinical notes. Type and date of surgery procedure is noted.

### Data Validation

Several validation techniques have been established to ensure complete case capture. Firstly, surgical logbooks in each centre (where each operation is recorded in detail) are examined on a regular basis to determine all cases that underwent vitreo-retinal surgery of any type. This list is then cross referenced with collected data during this time period to identify any potentially missed cases. Subsequently, all out-patient and discharge letters are viewed to determine how many cases were not recruited during the short time period.(e.g. one week) Any ambiguous or incomplete information is followed up by examination of individual clinical case-notes or by contacting the patient or surgeon directly. Cases that were missed in hospital are invited for inclusion at the first follow up appointment. Each nominated local investigator is also asked to record information on any willing participant who did not undergo surgery for medical or other reasons. Through these rigorous techniques we are confident in achieving a high case ascertainment level.

### Assessing under-ascertainment

Using a capture-recapture methodology, we will estimate the level of under-ascertainment in our study in order to obtain more accurate approximation of disease incidence. Capture-recapture methodology aims to adjust for the extent of incomplete ascertainment using information from overlapping lists of cases from distinct sources[25]. The information services division (ISD) has been established for over 40 years in Scotland and is a national organisation for health information and statistics. It comprises detailed data on all hospital and out-patient activity on a monthly basis across Scotland. All diagnostic and discharge data in Scotland is recorded and classified according to the International Classification of Disease (ICD) criteria. This data is accessible through an oversight committee and is part of NHS National Services Scotland. By comparing this data with data generated by our study we will conduct a two-source capture-recapture study to calculate the maximum likelihood estimate of ascertainment between the databases and provide a more accurate estimation of true disease incidence.

#### Statistical analysis

We will calculate standard error estimates and the 95% confidence intervals for all categorical and continuous data. Annual age and sex standardised incidence rates will be calculated. The prevalence rate and association of refractive error, peripheral retinal degeneration, and previous intra-ocular surgery with RRD in our study population will be calculated and presented. The analysis of the genome wide association study will be based on one-locus association tests with two degrees of freedom in order to avoid assumptions about the locus genetic model.

### Examination protocol

#### Visual acuity

Visual acuity at presentation is performed by a trained nurse practitioner using a Snellen chart at 6 meters. Best corrected visual acuity is measured separately for each eye and is recorded as the smallest line which the patient can correctly read. If the largest letter is not visualised (6/60), the examiner tests finger counting at one meter. The examiner will then test 'hand motion' detection at one meter and finally light perception is tested by with a pen-torch light and a visual acuity of perception of light, or no perception of light is then recorded. Focimetry is also performed on all participants who wear glasses.

#### Slit lamp examination

A slit lamp examination is performed on all participants. After appropriate positioning of the patient, the anterior segment of each eye is examined and the structures of the anterior segment are inspected for any lesions or abnormalities which are then documented.

#### Applanation tonometry

Intra-ocular pressure is measured by a standard method using a Goldmann Applanation Tonometer.

#### Dilated fundal examination

All participants have both eyes dilated and a systematic examination of both fundi is performed by a consultant ophthalmologist. Examination is performed initially at the slit-lamp using a condensing lens and subsequently using the indirect ophthalmoscope with scleral indentation. Each quadrant is examined thoroughly and all noted pathology is recorded on a standardised fundal drawing.

### Definitions

#### Type of RRD

Three conditions must be satisfied to cause RRD: vitreous liquefaction, tractional forces producing a retinal break and an open retinal break allowing fluid to gain access to the sub-retinal space.[26] The subtype of RRD is described according to the type of retinal break causing the detachment and may be classified as follows:

##### Horse-shoe shaped tear

This refers to tear in the neurosensory retina (NSR) due to a persistent site of vitreo-retinal traction during a posterior vitreous detachment.

##### Giant Retinal Tear

These are defined as circumferential retinal breaks of 90 degrees or more. The tear is usually along the posterior margin of the vitreous base, may have a rolled edge and rarely may occur at the anterior margin in the ciliary epithelium [27].

##### Dialysis

Defined as circumferential tears along the ora serrata with persistent vitreous attachment to the posterior retina (ie. No posterior vitreous detachment) [28].

##### Round Hole

This refers to an avulsed or operculated area of peripheral retina induced by a complete or localised vitreous separation. It may also refer to atrophic retinal holes associated with areas of pigmentation or peripheral retinal degeneration.

##### Retinoschisis

This describes a degenerative process causing a splitting of the retinal layers. If retinal breaks occurs in both the outer and inner leaves of a retinoschisis causing a progressive extension of sub-retinal fluid requiring intervention this is classified as a retinoschisis detachment [29].

### Ocular features defined

A myopic refractive error is defined as a spherical equivalent refractive error of<-1 dioptre (D), a hypermettropic refractive error as a spherical equivalent refractive error of >+1 D and emmetropia as a spherical equivalent refractive error of between -1 and +1 D.

Trauma is defined as any direct or indirect trauma to the eye resulting in loss or disturbance of vision warranting ophthalmological review.

Posterior vitreous detachment is due to progressive liquefaction of the aging vitreous which induces its separation from the inner-limiting membrane of the retina. This process may be partial or complete (often seen as a Weiss ring with no detectable adhesions around the posterior pole).[30]

Peripheral retinal degeneration delineates peripheral retinal lesions which cause anomalies in vitreo-retinal adhesion which may predispose to RRD.

Lattice degeneration is characterised clinically as circumferentially distributed, sharply demarcated, elliptical areas of peripheral retinal thinning with variable pigmentation. There are often localised areas of overlying vitreous liquefaction, a criss-crossing network of hyalinised vessels and exaggerated vitreoretinal attachments along the edge of the lesion [31].

### Ethical aspects

Multi-centre research ethics committee (Scotland) approval and NHS Trust management committee approval have been obtained from all participating sites. All participants included in the study are fully informed about the study. Participation is voluntary, individuals may withdraw at any stage and participation does not affect the treatment of the individual. All data is recorded and stored in compliance with ethical and data protection guidelines.

## Discussion

This study describes a prospective population based observational design which aims to accurately characterise the incidence and clinical associations of RRD in a well defined population. Findings from this study will represent a first estimate incidence of RRD in Scotland and will highlight the healthcare burden and distribution of this potentially blinding condition. This study will also provide an important genetic resource for the investigation of the genetic contribution to the aetiology of this condition.

## Competing interests

The authors declare that they have no competing interests.

## Authors' contributions

DM is responsible for overall recruitment and management of the project and drafted the manuscript. DC, DY, BF, JS are responsible for the design of the study, the study protocol and the co-ordination of resources. HC and AW are responsible for the design and coordination of the genetic database and will lead the statistical analysis. All authors read and approved the final manuscript.

## Pre-publication history

The pre-publication history for this paper can be accessed here:


